# What's new in Emergencies, Trauma, and Shock? Nitrogen balance in critical patients on enteral nutrition

**DOI:** 10.4103/0974-2700.62099

**Published:** 2010

**Authors:** Luigi Beretta, Simona Rocchetti, Marco Braga

**Affiliations:** Anesthesia and Neurointensive Care Unit, IRCCS S. Raffaele – Via Olgettina, 60 – 20132 Milano, Italy; 1 Department of Surgery, IRCCS S. Raffaele – Via Olgettina, 60 – 20132 Milano, Italy

After insults such as shock, trauma, burns, and sepsis, the resulting hypermetabolism and catabolism can cause malnutrition. A major goal of modern clinical nutrition is to modify stress response below the extreme and thereby positively influence recovery. Optimum nutrition support with amino acids should be adjusted to the underlying metabolic state of the patient.

The care of injured patients has improved over the past decade. The factors responsible for this include effective management of shock and fluid/electrolyte balance, as well as the availability of new means of nutritional support.

The improved efficacy of providing substrate to the injured, immunocompromised, or malnourished host has caused a renaissance in the clinical application of dietetics in the treatment and prevention of disease.[1] Presently, it is feasible to employ specialized nutritional support and to modify the metabolic response to stress by using pharmacologic doses of specific nutrients, especially those related to protein metabolism. Indeed, aggressive nutritional support plays a major role in the comprehensive care of injured or critically ill patients.[2]

After a variety of insults (shock, trauma, burns, sepsis, pancreatitis, etc.), patients develop a systemic inflammatory response that is presumably beneficial and resolves as the patient recovers. However, if the systemic inflammatory response is exaggerated or perpetuated, severe disturbances in protein metabolism may arise. The resulting hypermetabolism and catabolism can cause acute protein malnutrition, with impairment in immune function and subclinical multiple organ dysfunction, including acute renal failure.[3]

The hypermetabolism of the stressed patients – whether from injury or infection – is associated with increases in muscle proteolysis, hepatic ureagenesis associated with enhanced glucose production, and increased mobilization of fat. Both injured and septic patients reveal an increased rate of whole-body protein catabolism with a slight increase in protein synthesis, leading to a negative nitrogen (N) balance. Infusion with an adequate amount of amino acids partially improves the synthesis rate, but the extent of protein catabolism is insensitive to intravenous nutrition.[4]

The metabolic rate and N excretion are related to the extent of injury. The two responses generally parallel each other. It has been demonstrated that the infusion of a combination of cortisol, epinephrine, and glucagon could increase the glycemia seen in stress conditions and also increase thermogenesis and N loss.[5]

The cascade of alterations in neuroendocrine control mechanisms resulting from critical illness profoundly influences protein and amino acid metabolism and, thus, body protein components. When starvation is superimposed on injury or critical illness, the metabolic alterations commonly associated with starvation that allow for a reduction in energy expenditure and protein sparing do not occur. Instead, lean body mass is catabolised as an energy source to meet the increased energy needs. Following an acute injury without a low-flow state, lean body mass is first mobilized and then lost. Loss of lean body mass or, more specifically, body cell mass can result in impaired host defence and increase morbidity and mortality in critical illness.[6]

In critically ill patients, up to 20% of body proteins are lost within 3 weeks, mostly during the first 10 days following injury; about 70% of this protein loss comes from skeletal muscle.[7]

Estimations of N loss can be obtained from urine urea nitrogen (UUN) and approximation of nonurinary N losses. In the critically ill patient, abnormal N losses may occur through burn wound exudate, fistula drainage, gastrointestinal fluid loss, diarrhea, or dialysis. Measurement of 24-h UUN excretion has been used to evaluate the degree of catabolism. When used to determine the degree of stress, the most sensitive and specific measure of N excretion is obtained in the fasting state.

When there is excessive muscle wasting associated with increased whole-body energy expenditure-as in severe injury, after burns of more than about 10% of body surface area,[8–10] during infection,[11] and in some kind of cancer[12,13] – whole-body N balance is markedly negative, probably because of changes that include an increase of protein breakdown in different tissues. Whether muscle protein synthesis also falls, as might be expected, or rises (possibly as an adaptive response to the rise in protein breakdown) is not known. The major change is a marked acceleration of lean tissue proteolysis,[14] probably including muscle proteolysis. The activation of muscle proteolysis can be evaluated by measuring urinary excretion of 3-methylhistidine, which is increased in injured patients. In standardized conditions, negative N balance is 8 times higher in patients with severe burns and 6 times higher in those with severe injury than in normal subjects. In severe traumatic conditions, urinary excretion of N may reach 35–40 g per day, the equivalent of more than 1 kg lean body mass.[15]

N balance reflects the difference between whole-body protein synthesis and breakdown. N balance studies are best used not to determine nutritional status *per se* but to determine whether nutrition support has been sufficient to prevent net catabolism or to promote anabolism. It was established that an improved N balance could be approached in severe catabolic states by increasing N provision, though the augmented N loss is not reduced by the N intake.[16]

The patient whose problem is primarily partial starvation can be put into positive N balance with good nutritional support, whereas the strongly catabolic patient cannot achieve a positive N balance by nutritional intervention until the peak of the catabolic drive has passed. Kinetic studies indicate that the provision of a protein intake of up 1.5 g/kg/day can improve the N balance, but going above that level of intake merely increases the rate of protein synthesis and breakdown without improving the N balance.[17] Indeed, current forms of nutrition support are relatively inefficient in stimulating protein synthesis or in reducing protein breakdown during critical illness, especially in muscle.

Recent patterns show that critical illness or endocrine disease, which result in lean tissue wasting over a long period of time, also depress muscle protein turnover through reduced protein synthesis and protein breakdown. Whole-body protein turnover is, therefore, also depressed, although in some circumstances visceral protein turnover may be elevated (e.g., liver and kidney disease). Under these circumstances, it make sense that any attempts to replenish depleted cell mass should aim to increase muscle protein synthesis rather than to decrease muscle protein breakdown.[4]

In traumatized patients, the response of N balance and 3-methylhistidine excretion to exogenously administered amino acids is of interest. Parenteral nutrition with adequate levels of amino acids and energy markedly improves N balance as compared to administration of energy substrates alone. Measurement of 3-methylhistidine excretion under each of these conditions, however, indicates no difference in skeletal muscle catabolic rates.[18] These data suggest that exogenous nutritional support is effective in increasing protein synthesis in the presence of increased protein catabolism and that increased protein synthesis results in improved N balance.

The development of clinical nutrition reveals a remarkable picture. Increased N loss associated with hypermetabolism attracted scientific attention because of the new interest in adrenocortical hormones and the cortisol secretion induced by injury, which causes muscle breakdown and a negative N balance.[19]

A major goal of modern clinical nutrition is to modify stress response below the extreme and thereby positively influence recovery. Optimal nutrition support with amino acids should be adjusted to the underlying metabolic state of the patient. The metabolic state is the result of some blend of the response to starvation and the response to injury, infection, or a specific disease. In all cases, adequate energy and N substrates are provided to meet the increased requirements of catabolic, hypermetabolic, or depleted patients. Improved understanding of regulatory mechanisms may lead to novel therapies that could modify the intensity or nature of the injury response, thus altering the consequent metabolic demands.

The current guidelines on nutritional intervention in critically ill patients recommend the utilization of enteral nutrition (EN) in all ICU patients who are not expected to take a full oral diet within 3 days. EN should begin during the first 24 h, using a standard high-protein formula.[20] Opinion leaders in the field of EN consider the immunologic benefits of EN more important than the delivery of calories or improvement of N balance. The proposed advantages of EN in surgical and critically ill patients are now well described. They include attenuation of the metabolic response to stress, improved N balance, better glycemic control, increased visceral protein synthesis, enhanced gut oxygenation, and increased collagen deposition.[21–30] Other beneficial effects include decreased rate of nosocomial infections[31] and enhanced visceral blood flow.[32,33] The enhanced splanchnic blood flow due to EN seems to allow a better utilization of both endogenous and administered amino acids, with a lower N shift from the muscle to the liver and a consequent lower tendency to negative N balance.

The pattern of changes in amino acid concentration in muscle during catabolism shows an increase in branched-chain amino acids, aromatic amino acids, and methionine, and a decrease in glutamine and basic amino acids (lysine and arginine). A uniform reduction of approximately 50% of free muscular glutamine associated with negative N balance seems to be one of the most typical features of the response to trauma and infection.[34] The marked intracellular glutamine depression has been demonstrated after elective operation, major injury, burns, infections, and pancreatitis, irrespective of nutritional attempts at repletion.

Reduction of the muscle free glutamine pool thus appears to be a hallmark of the response to injury, and its extent and duration is proportional to the severity of illness. Recent studies have underlined that the tissue glutamine depletion is caused mainly by stress-induced alterations in the interorgan glutamine flow.[35] Muscle, and probably lung glutamine effluxes, are accelerated to provide substrate for the gut, immune cells, and kidneys,[36] explaining at least in part the profound decline in muscle free glutamine concentration. During catabolic stress, peripheral glutamine stores are rapidly diminished and the amino acid is preferentially shunted toward visceral organs as a fuel source. This creates a glutamine-depleted environment, with subsequent enterocyte and immunocyte starvation.[37] Consequently, glutamine is considered a conditionally essential amino acid and should be administered during episodes of catabolic stress. Many clinical investigations have shown improved N economy and maintained glutamine concentration with glutamine or glutamine-containing dipeptide supplementation. Glutamine should be added to a standard enteral formula in burn and trauma ICU patients.
